# Niche modeling for the genus *Pogona* (Squamata: Agamidae) in Australia: predicting past (late Quaternary) and future (2070) areas of suitable habitat

**DOI:** 10.7717/peerj.6128

**Published:** 2018-12-17

**Authors:** Julie E. Rej, T. Andrew Joyner

**Affiliations:** 1 Department of Wildlife Ecology, The Wilds, Cumberland, OH, USA; 2 Department of Geosciences, East Tennessee State University, Johnson City, TN, USA

**Keywords:** *Pogona*, Agamidae, Late quaternary, Niche modeling, Future 2070, Squamate, Conservation, Climate change, MaxEnt, Australia

## Abstract

**Background:**

As the climate warms, many species of reptiles are at risk of habitat loss and ultimately extinction. Locations of suitable habitat in the past, present, and future were modeled for several lizard species using MaxEnt, incorporating climatic variables related to temperature and precipitation. In this study, we predict where there is currently suitable habitat for the genus *Pogona* and potential shifts in habitat suitability in the past and future.

**Methods:**

Georeferenced occurrence records were obtained from the Global Biodiversity Information Facility, climate variables (describing temperature and precipitation) were obtained from WorldClim, and a vegetation index was obtained from AVHRR satellite data. Matching climate variables were downloaded for three different past time periods (mid-Holocene, Last Glacial Maximum, and Last Interglacial) and two different future projections representative concentration pathways (RCPs 2.6 and 8.5). MaxEnt produced accuracy metrics, response curves, and probability surfaces. For each species, parameters were adjusted for the best possible output that was biologically informative.

**Results:**

Model results predicted that in the past, there was little suitable habitat for *P. henrylawsoni* and *P. microlepidota* within the areas of their current range. Past areas of suitable habitat for *P. barbata* were predicted to be similar to the current prediction. *Pogona minor* and *P. nullarbor* were predicted to have had a more expansive range of suitable habitat in the past, which has reduced over time. *P. vitticeps* was predicted to have less suitable habitat in the past when examining the region of their known occurrence; however, there was predicted growth in suitable habitat in Western Australia. Both 2070 models predict a similar distribution of habitat; however, the model produced using the 2070 RCP 8.5 climate change projection showed a larger change, both in areas of suitable habitat gain and loss. In the future, *P. henrylawsoni* and *P. microlepidota* might gain suitable habitat, while the other four species could possibly suffer habitat loss.

**Discussion:**

Based on the model results, *P. henrylawsoni* and *P. microlepidota* had minimal areas of suitable habitat during the Last Glacial Maximum, possibly due to changes in tolerance or data/model limitations, especially since genetic analyses for these species suggest a much earlier emergence. The predicted late Quaternary habitat results for all species of *Pogona* are conservative and should be compared to the fossil record which is not possible at the moment due to the current inability to identify fossil *Pogona* to the species level. *P. nullarbor* and *P. vitticeps* future models predict substantial habitat loss. *P. nullarbor* could potentially be considered vulnerable in the present since it already has a restricted range, and a conservation plan may need to be considered.

## Introduction

Reptiles are ectothermic tetrapods that are currently threatened by several global problems such as habitat loss, invasive species, environmental pollution, disease and parasitism, unsustainable use, and rising global temperatures (Gibbons et al., 2000; Hoffmann, Chown & Clusella-Trullas, 2012). These threats appear to be of great danger to many reptile species and extinctions worldwide are expected; however, population declines can be difficult to detect (Gibbons et al., 2000). The limited dispersal abilities of reptiles cause them to be threatened by rapid environmental changes. Such is the case for many species of Australian lizards that are currently at risk of severe range restriction or even extinction (Brereton, Bennet & Mansergh, 1995; Gibbons et al., 2000). Models predict future habitat change for several Australian skink species, and climate-driven changes may cause the extinction of the many threatened species (Brereton, Bennet & Mansergh, 1995; Fordham et al., 2012).

Climate shifts have often occurred over Earth’s vast history; as a result, many organisms experience changes in morphology, physiology, geographic location, and/or extinction in response to the changes. The squamate fossil record has been the subject of limited studies, and the effects of climate on past squamate populations are even less known (Gray, Reed & McDowell, 2015). Currently, there is limited published work on the late Quaternary fossil history of Agamidae from Australia, and fossil remains of *Pogona* cannot be identified beyond the generic level with what is currently known about their skeletal morphology (Gray, Reed & McDowell, 2015; Gray et al., 2017; Rej, 2017); however, our ongoing research of agamid osteology may help inform which species are present at particular localities across Australia.

*Pogona*, commonly known as the bearded dragon, is a genus of dragon lizard (Agamidae: Squamata), that is, distributed across Australia (Wilson & Swan, 2013), and the effects of climate change on this group are yet to be examined. *Pogona* consists of six species and three (*P. barbata*, *P. minor*, and *P. vitticeps*) have an extensive range, while the other three (*P. henrylawsoni*, *P. microlepidota*, and *P. nullarbor*) have an extremely restricted range (Uetz, Freed & Hosek, 1995; Wilson & Swan, 2013). The only species of *Pogona* currently listed on the IUCN Red List is *P. barbata*, which is listed as a species of least concern (Hutchinson, 2010). The remaining five species have yet to be evaluated. With this project, we hope to examine the current range of suitable habitat for each species of *Pogona*, potential habitat shifts in response to climate change in the future, and the potential past locations of suitable habitat during the late Quaternary. Analyzing the current and predicted suitable habitat range of each *Pogona* species may identify vulnerable species that should be considered for conservation efforts (Brereton, Bennet & Mansergh, 1995).

Estimating past habitats may be helpful for paleontology and evolutionary studies (Jurestovsky & Joyner, 2017). We are interested in projecting potential past suitable habitat for two reasons. First, we are interested in examining overall trends in suitable habitat changes over a larger range of time. By examining the past projections, including periods that are cooler then today (mid-Holocene and Last Glacial Maximum) and periods that are warmer (Last Interglacial), we may be able to better interpret the future projections. Second, once fossil *Pogona* is identifiable to the species level, comparing the fossil localities with the past projections could potentially help with understanding past evolutionary changes in physiology and the bearded dragon’s ability to adapt to a changing environment, especially if the model does not match the fossil record.

**Figure 1:**
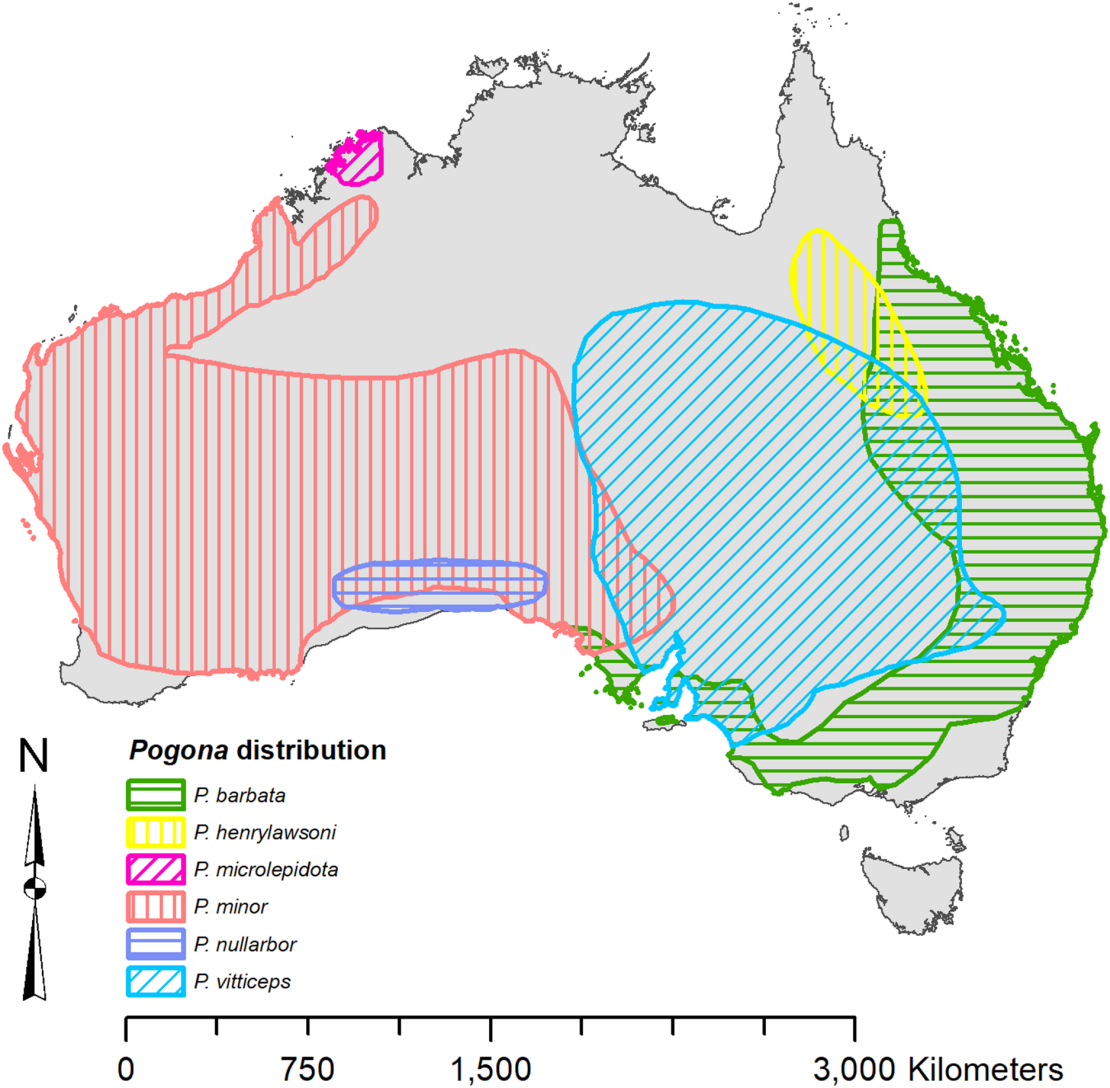
Known distribution of *Pogona* georeferenced from Wilson & Swan (2013).

Each species of *Pogona* has its own distinct range (Fig. 1) (Wilson & Swan, 2013). *Pogona barbata* is found in the east and prefers a woodland to dry sclerophyll forest habitat; however, *P. barbata* is commonly seen around cities. *P. henrylawsoni* is found in the inland north-central region of Queensland, and their habitat is a treeless cracked clay plain with Mitchell grass as the only vegetation. *P. microlepidota* is restricted to the northwest of Kimberley in Western Australia, and prefers a woodland habitat with a more humid climate. *P. minor* is found in the west and prefers a woodland to shrubland habitat. *P. nullarbor* is restricted to the Nullarbor Plain and prefers a shrubland habitat with minimal to moderate humidity. *P. vitticeps* is found throughout the eastern interior of Australia, and it prefers a semi-arid to arid woodland habitat with low humidity.

In this study, we used MaxEnt (maximum entropy) v3.3.3 to predict past, current, and future habitat ranges for *Pogona*. For our projected models into the past and future, we are assuming that tolerance is the same as the modern species. MaxEnt is a general-purpose machine learning method, that is, well-suited for species distribution modeling (Phillips, Anderson & Shapire, 2006). A higher area under the curve (AUC) value indicates better discrimination between suitable and unsuitable habitat predictions for species (Elith et al., 2006). MaxEnt only requires presence data, which is helpful since absence data are not common and are much more difficult to verify. Additionally, MaxEnt has been used in several previous studies to model other lizard groups across the globe for conservation purposes. In previous studies, climate variables relating to temperature and precipitation were used since they affect thermo-regulation and metabolism in reptiles. In this study, we use similar climatic variables.

## Materials and Methods

**Figure 2:**
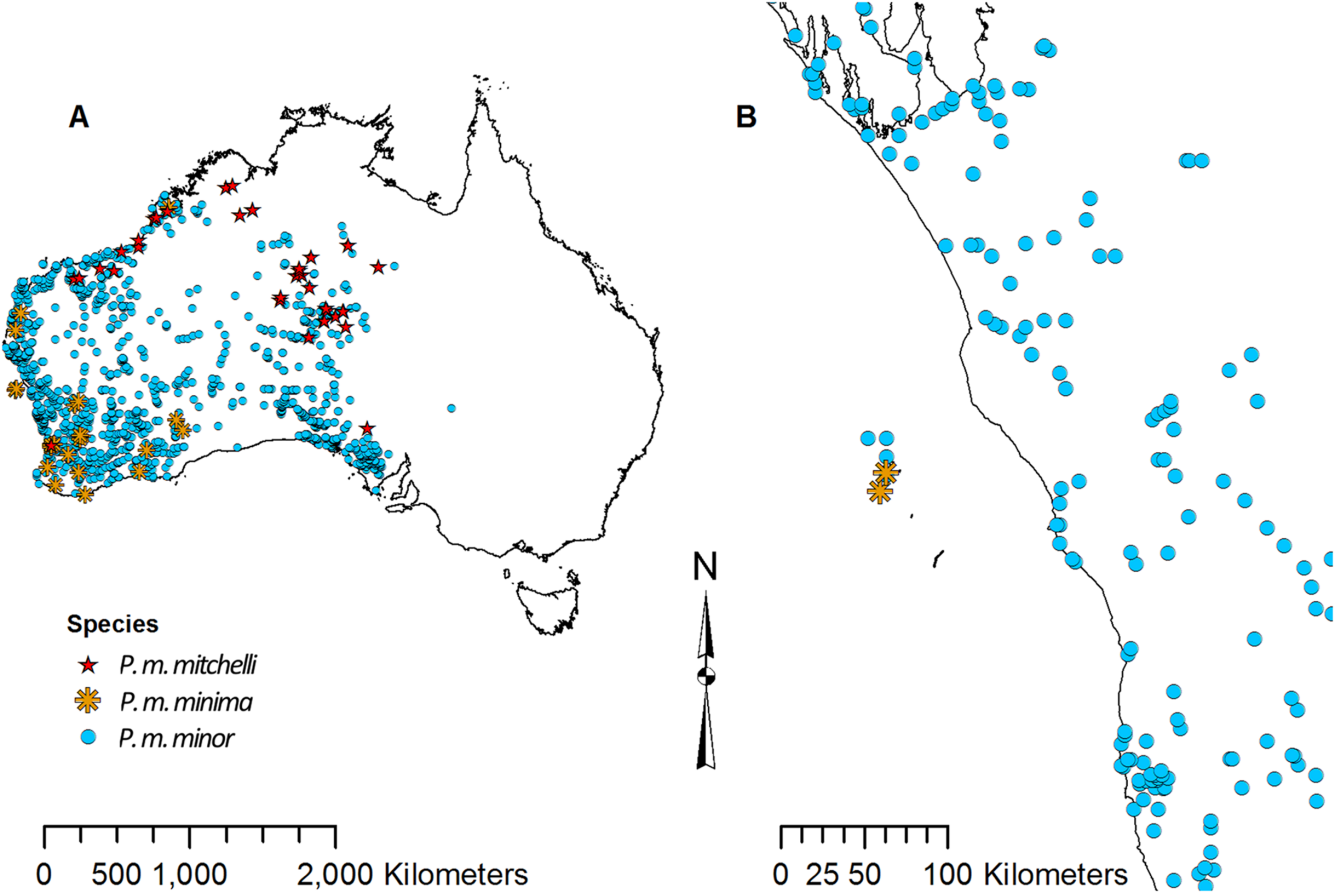
Point data for *Pogona minor mitchelli*, *P. m. minima*, and *P. m. minor*. (A) Distribution across Australia. (B) Distribution on Houtman Abrolhos islands and the lower west coast of Western Australia.

Point data for each species of *Pogona* were downloaded from the Global Biodiversity Information Facility (GBIF) (GBIF, 2016). There are currently six recognized species; however, *P. minor* consists of three subspecies: *P. minor minor*, *P. minor minima*, and *P. minor mitchelli* (Wilson & Swan, 2013). Some literature considers *P. m. minima* and *P. m. mitchelli* to be separate species (Uetz, Freed & Hosek, 1995), and as a result, the point data that were available for these three subspecies of *P. minor* were slightly ambiguous. *P. m. minima* is restricted to the Houtman Abrolhos islands off the coast of Western Australia, *P. m. mitchelli* is found north of the Pilbara, and *P. m. minor* is found throughout the west, but does not overlap with *P. m. minima* or *P. m. mitchelli* (Wilson & Swan, 2013); however, the point data showed overlap (Fig. 2). Due to this overlap, *P. m. minor* data clearly contained *P. m. minima* and *P. m. mitchelli* records. For this study we did not analyze *P. m. minima* or *P. m. mitchelli* separately since their point data did not match the known distribution. Also, since *P. m. minor*’s point data overlapped with the other two, we considered the *P. m. minor* data to have contained points for all three subspecies, and we referred to the *P. m. minor* data collectively as *P. minor* in this study.

We removed points with no coordinates as well as points well beyond the known occurrence for each species. This resulted in 3,357 *P. barbata*, 34 *P. henrylawsoni*, 29 *P. microlepidota*, 2,548 *P. minor*, 105 *P. nullarbor*, and 3,826 *P. vitticeps* occurrence points. Using the Species Distribution Model (SDM) toolbox in ArcMap 10.4 (Esri, Redlands, CA, USA), each set of species point data was spatially rarefied to one km (Brown, 2014). Rarefied points were then divided into training and testing points. The size of the training feature subset was set to 80%, which resulted in a 20% testing subset.

All climate data were downloaded from WorldClim (Hijmans et al., 2005). Current bioclimatic variables were downloaded at 30 arc seconds (approximately one km spatial resolution) and at 2.5 min (approximately five km spatial resolution). Future bioclimatic variables for the year 2070 were downloaded from WorldClim at one km from the CCSM4 global climate/circulation model (GCM) for representative concentration pathways (RCPs) 2.6 and 8.5. There are several different future predicted climate scenarios for 2070 based on greenhouse gas emissions (IPCC, 2013). The RCP 2.6 projection represented the best-case scenario where emissions will peak by 2020 then decline over time, while the RCP 8.5 projection represented the worst-case scenario in which gas emissions will continue to rise (IPCC, 2014). By selecting both, we projected future suitable habitat, considering either scenario and spanning the range of expected future climate change. Past bioclimatic variables for mid-Holocene (∼6,000 years ago), Last Glacial Maximum (∼21,500 years ago), and Last Interglacial (∼130,000–116,000 years ago) were also downloaded from WorldClim (Hijmans et al., 2005). Both mid-Holocene and Last Glacial Maximum data were downloaded from the CCSM4 GCM but at different resolution due to availability. Only the mid-Holocene was available at one km and the finest resolution available for Last Glacial Maximum was five km. Last Interglacial climate data were available from one model type and had a resolution of one km (Otto-Bliesner et al., 2006).

For comparison, we downloaded past (mid-Holocene and Last Glacial Maximum) and future (2070 2.6 and 8.5 RCP) climate data from the MIROC earth system model (ESM) and the MPI ESM from WorlClim (Hijmans et al., 2005). Similar to the CCSM4 GCM data, resolution was one km for all data with the exception of Last Glacial Maximum which was downloaded at five km. In MaxEnt, models were developed for each of the three GCM’s (CCSM4 GCM, MIROC ESM, and MPI ESM) and the outputs were compared. The three GCM’s selected were the only downscaled and bias-corrected GCM’s available from WorldClim across all time periods (which include past and future climate data).

For this study we selected six climatic variables: annual mean temperature (Bio 1), maximum temperature of the warmest month (Bio 5), minimum temperature of the coldest month (Bio 6), annual precipitation (Bio 12), precipitation of the wettest quarter (Bio 16), and precipitation of the driest quarter (Bio 17). We selected the three temperature variables because a reptile’s activity level and health are dependent on their overall body temperature (Cannon, 2003). When active, the optimal body temperature for *Pogona* ranges from 35 to 39 °C, and to maintain this body temperature *Pogona* will display a variety of behaviors to either heat up or cool down (Cannon, 2003; Doneley, 2006). If the *Pogona*’s body temperature falls too low, their risk of disease is greatly increased, and they cannot digest food properly (Cannon, 2003); however, in the winter months the daytime temperatures can drop to below 10 °C in the southern regions of Australia, resulting in brumation behavior, which is not only normal but a necessary stimulant for reproduction in early spring (Doneley, 2006). We also selected three precipitation variables because *Pogona* requires a very precise amount of humidity in their environment. Overall, *Pogona* is adapted to an arid and dry environment (Cannon, 2003), but species are exposed to seasonal rains and spend time in humid microhabitats (Wright, 2008). *Pogona* can develop a respiratory infection if exposure to humidity higher than 40% is prolonged (Doneley, 2006), but without moisture in the environment, dehydration will occur, causing improper nutrient absorption and constipation (Wright, 2008).

Additionally, normalized difference vegetation index (NDVI) was included for comparison with a model using only the six climatic variables (Hay et al., 2006). A global NDVI variable was developed at ∼1 km resolution by the Trypanosomiasis and Land use in Africa research group using multi-year averaged AVHRR satellite data and made available upon request. Since vegetation coverage cannot be projected into the future or past, a comparison was done with and without NDVI. If the maps were similar, then the climate variables were considered a good predictor of occurrence in the past and future.

In MaxEnt, several test models were developed to find the best combination of parameters for each species before projecting into the past and future. Parameters modified followed those proposed by Merow, Smith & Silander (2013) as being more biologically informative and included bias correction, background data selection, regularization value selection, and output type. Before developing the MaxEnt models, both coordinate and sampling biases were corrected. To correct for coordinate (latitudinal) bias, we projected all data points (pre-rarefied) and variable rasters to an equal area projection, since geographic coordinate systems contain areal changes moving from the equator to the poles (Elith et al., 2011). To correct for sampling bias, we used the Gaussian kernel density of sampling localities tool from the SDM toolbox on each species’ set of rarefied points. The result is a reduction of the area where background points are sampled, with only areas within or near (∼55.66 km or 0.5 decimal degree, with some variance between species given the distance decay of the underlying density surface) current species locality records utilized for background sampling. The density estimation surface is a weighted surface based on the number of occurrence records per unit area and was used to correct for sampling bias when running the models. In MaxEnt, all samples were added as background points and point data only included localities where the species was likely to inhabit. Different regularization values were tested in MaxEnt for each species and selected based on AUC values and overall range output. We tested values 0.5, 1, 3, and 5, as suggested by Brown, Bennett & French (2017). Higher regularization values (i.e., >1.0) are sometimes needed to reduce overfitting caused by lower regularization multiplier values (Radosavljevic & Anderson, 2014). While we strived for the highest AUC, selecting a value that had a model output most accurate to the species known range was also important. Last, we selected raw output in MaxEnt since it does not rely on post-processing assumptions and is the best output type for projecting into the past and future (Merow, Smith & Silander, 2013).

**Figure 3:**
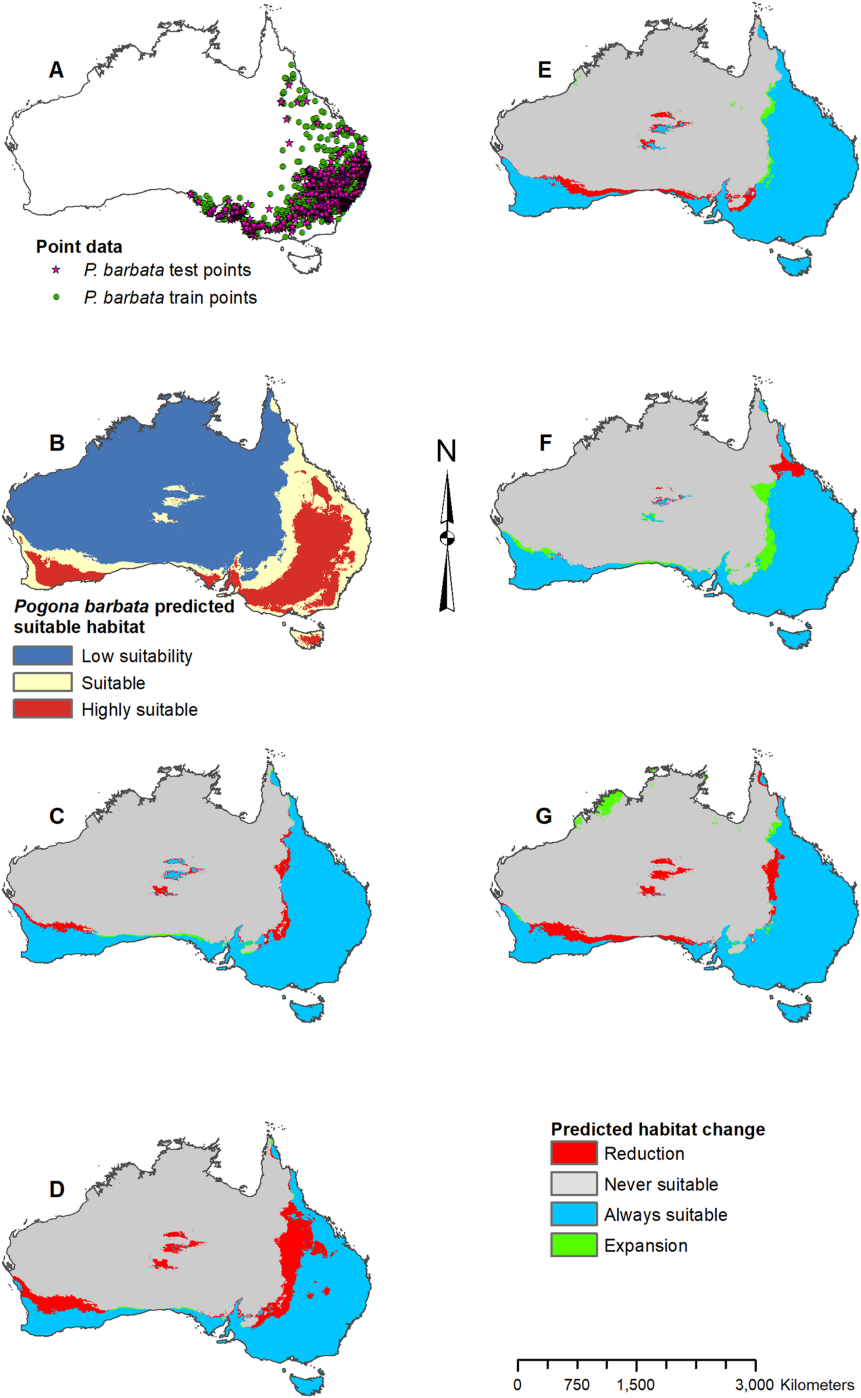
Result maps for *Pogona barbata*. (A) Test and train point distribution. (B) Current predicted areas of suitable habitat where highly suitable is >39.9% of occurrences predicted, and low suitability is <16.9% of occurrences predicted. (C) Future (2070 RCP 2.6) predicted change. (D) Future (2070 RCP 8.5) predicted change. (E) Past (mid-Holocene) predicted change. (F) Past (Last Glacial Maximum) predicted change. (G) Past (Last Interglacial) predicted change.

**Figure 4:**
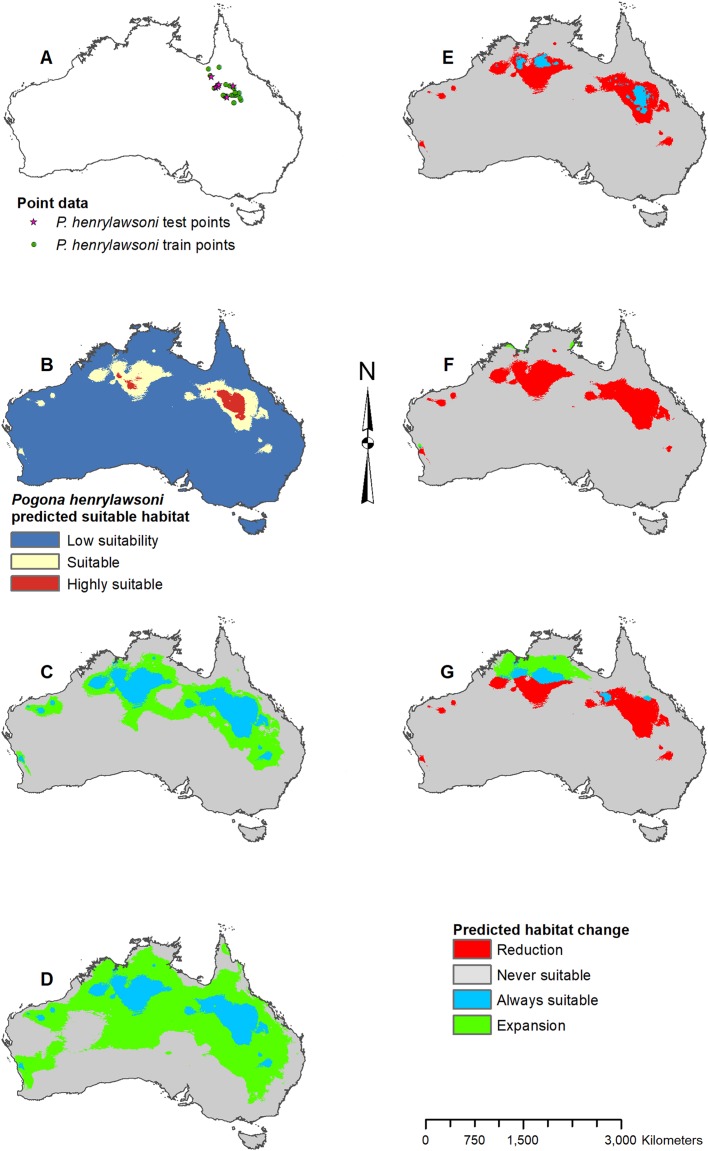
Result maps for *Pogona henrylawsoni*. (A) Test and train point distribution. (B) Current predicted areas of suitable habitat where highly suitable is >30.2% of occurrences predicted, and low suitability is <7.8% of occurrences predicted. (C) Future (2070 RCP 2.6) predicted change. (D) Future (2070 RCP 8.5) predicted change. (E) Past (mid-Holocene) predicted change. (F) Past (Last Glacial Maximum) predicted change. (G) Past (Last Interglacial) predicted change.

**Figure 5:**
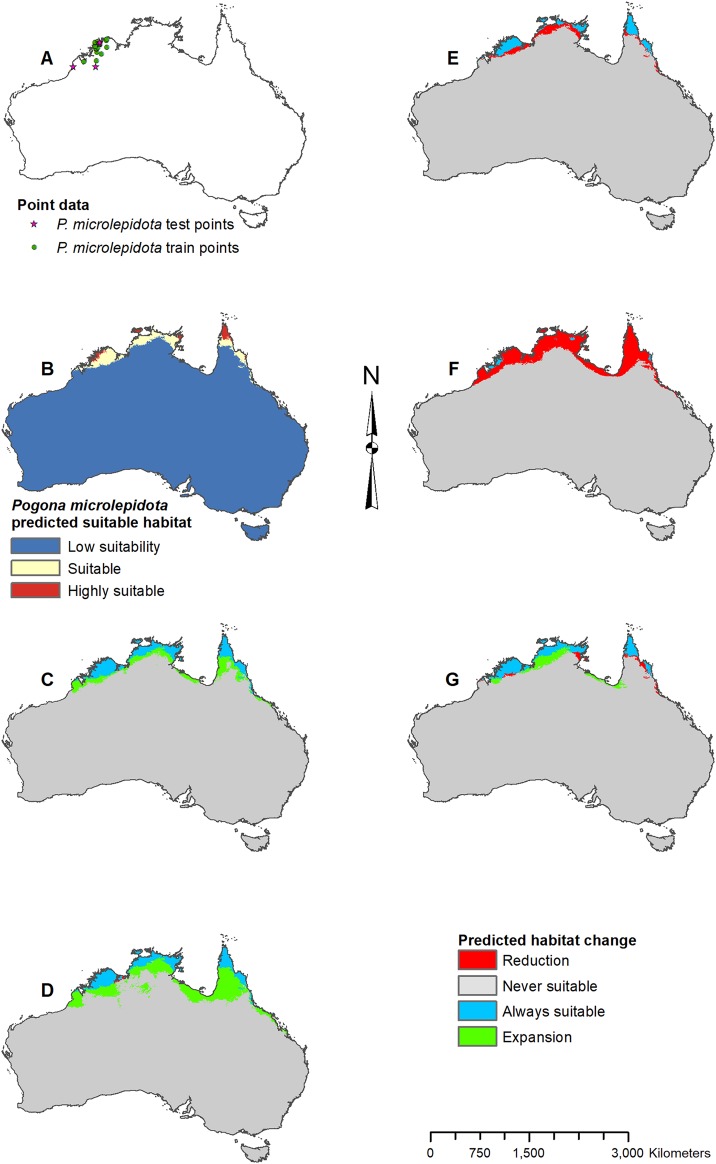
Result maps for *Pogona microlepidota*. (A) Test and train point distribution. (B) Current predicted areas of suitable habitat where highly suitable is >34.9% of occurrences predicted, and low suitability is <8.6% of occurrences predicted. (C) Future (2070 RCP 2.6) predicted change. (D) Future (2070 RCP 8.5) predicted change. (E) Past (mid-Holocene) predicted change. (F) Past (Last Glacial Maximum) predicted change. (G) Past (Last Interglacial) predicted change.

**Figure 6:**
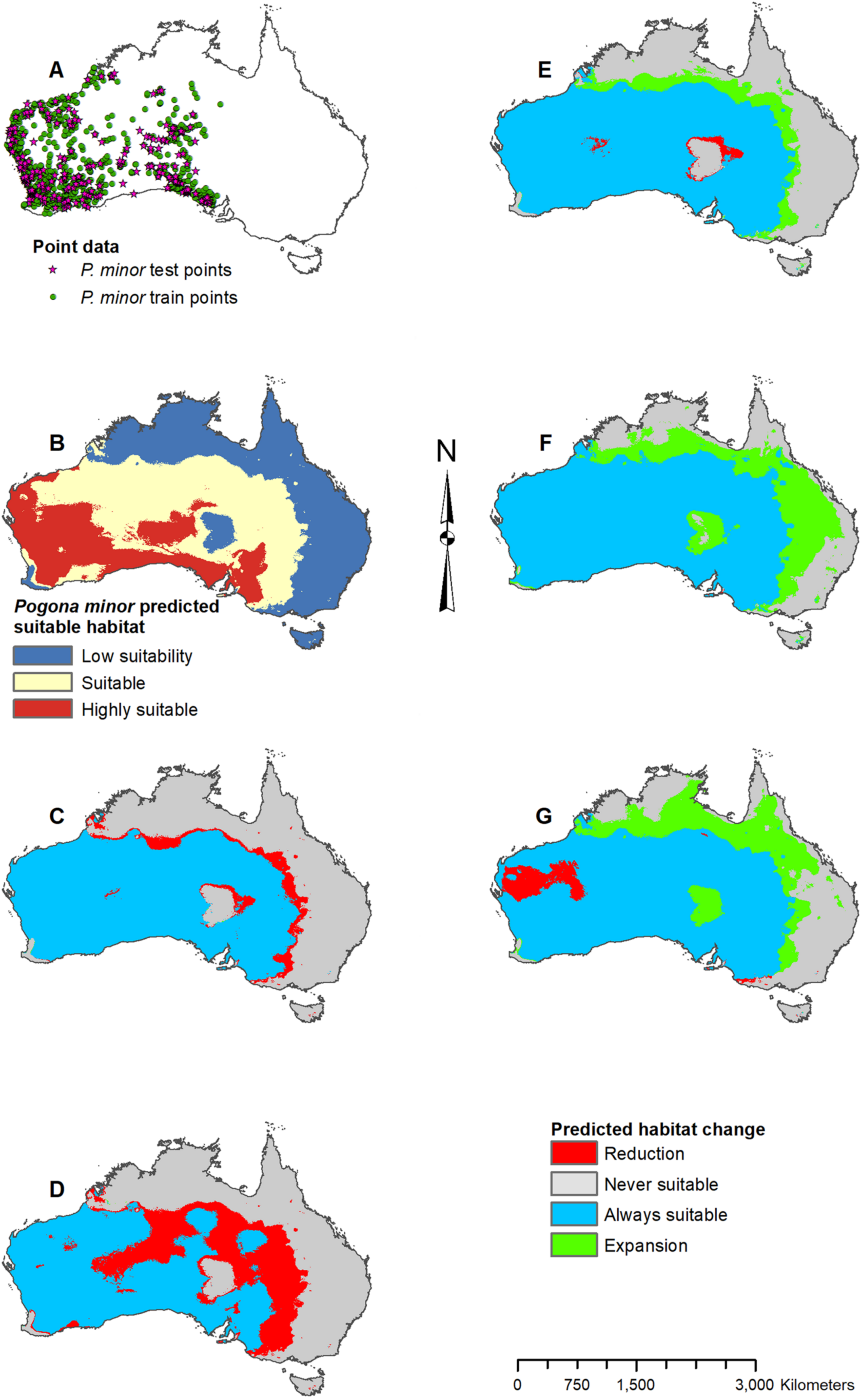
Result maps for *Pogona minor*. (A) Test and train point distribution. (B) Current predicted areas of suitable habitat where highly suitable is >28.6% of occurrences predicted, and low suitability is <14.9% of occurrences predicted. (C) Future (2070 RCP 2.6) predicted change. (D) Future (2070 RCP 8.5) predicted change. (E) Past (mid-Holocene) predicted change. (F) Past (Last Glacial Maximum) predicted change. (G) Past (Last Interglacial) predicted change.

**Figure 7:**
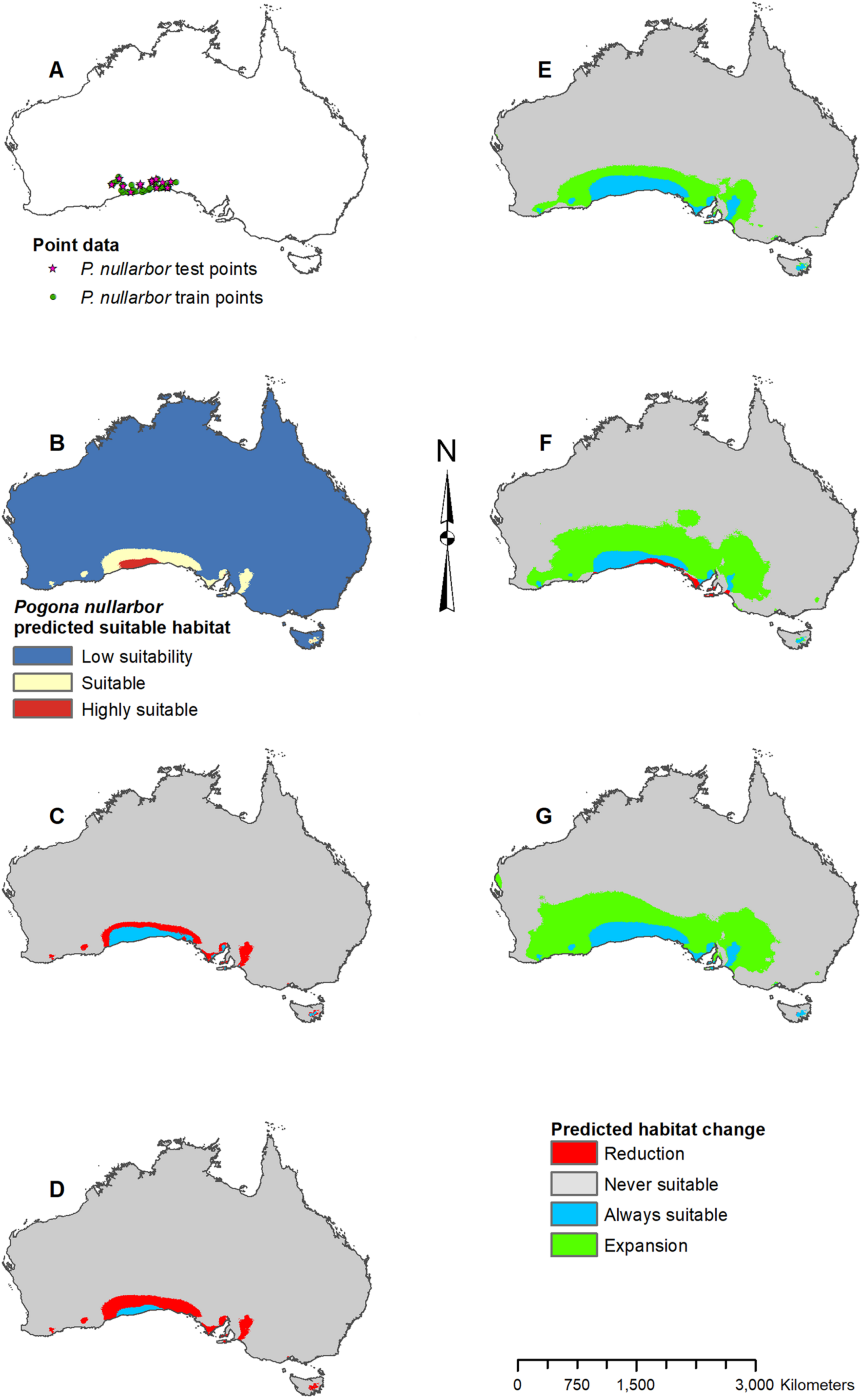
Result maps for *Pogona nullarbor*. (A) Test and train point distribution. (B) Current predicted areas of suitable habitat where highly suitable is >25.1% of occurrences predicted, and low suitability is <5.5% of occurrences predicted. (C) Future (2070 RCP 2.6) predicted change. (D) Future (2070 RCP 8.5) predicted change. (E) Past (mid-Holocene) predicted change. (F) Past (Last Glacial Maximum) predicted change. (G) Past (Last Interglacial) predicted change.

**Figure 8:**
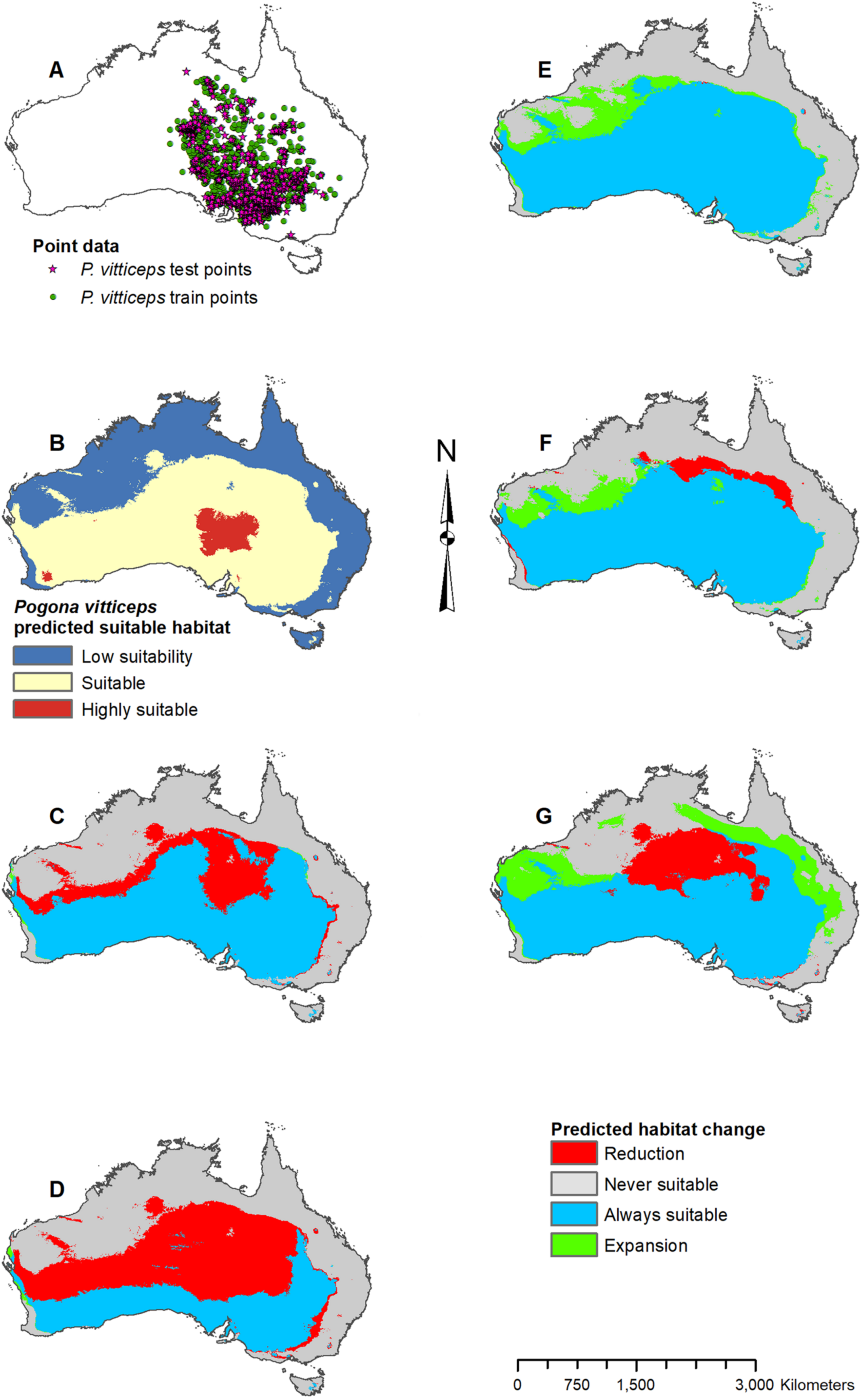
Result maps for *Pogona vitticeps*. (A) Test and train point distribution. (B) Current predicted areas of suitable habitat where highly suitable is >48.1% of occurrences predicted, and low suitability is <20.9% of occurrences predicted. (C) Future (2070 RCP 2.6) predicted change. (D) Future (2070 RCP 8.5) predicted change. (E) Past (mid-Holocene) predicted change. (F) Past (Last Glacial Maximum) predicted change. (G) Past (Last Interglacial) predicted change.

In addition to adjusting parameters, response curves and variable jackknifes were created during the modeling process to assess overall variable importance and the range of variable influence. Once the parameters were determined for each species, five projection models were developed that predicted two future (2070 RCP 2.6 and RCP 8.5) and three past (Last Interglacial, mid-Holocene, and Last Glacial Maximum) climate scenarios. Original model projections of the two future time periods, mid-Holocene, and Last Glacial Maximum were developed using the CCSM4 GCM data. Subsequent models were developed using the MIROC ESM and the MPI ESM to compare with the CCSM4 GCM results. With all models complete, the predicted occurrences were then imported into ArcMap for final analysis and map processing. Maps were converted to binary and the raster calculator tool was used to find areas of suitable habitat gain and loss. Suitability thresholds were derived from natural data breaks, which varied by model and species based on logistic threshold output from MaxEnt. All map figures were then finalized in ArcMap, and the percentages listed in Figs. 3–8 and Fig. S1 represent percent of occurrences predicted within each suitability level.

## Results

*Pogona* species with the larger ranges (*P. barbata*, *P. minor*, and *P. vitticeps*) had AUC values in the 0.70–0.79 range with high commission rates, while smaller ranged species (*P. henrylawsoni*, *P. microlepidota*, and *P. nullarbor*) had AUC values in the >0.9 range with low commission rates (Table 1). For both *P. barbata* and *P. vitticeps*, annual precipitation was the most important climatic variable when predicting suitable habitat, and both ranked maximum temperature during the warmest month and precipitation during the wettest quarter in their top three (Table 2). *P. henrylawsoni* and *P. nullarbor* both ranked maximum temperature during the warmest month as the most important variable for predicting habitat, followed by annual precipitation as the second most important (Table 2). *P. microlepidota* and *P. minor* selected annual temperature as the most important variable for predicting habitat, however, for *P. minor*, the value was much lower and precipitation during the driest quarter was nearly as important as annual temperature (Table 2).

**Table 1:** *Pogona* accuracy metrics.

	Training AUC	Testing AUC	Omission (%)	Commission (%)
*Pogona barbata*	0.773	0.772	1.2	40.6
*Pogona henrylawsoni*	0.970	0.981	8.3	8.7
*Pogona microlepidota*	0.984	0.958	6.2	6.3
*Pogona minor*	0.762	0.752	9.9	59.2
*Pogona nullarbor*	0.989	0.983	9.8	2.3
*Pogona vitticeps*	0.737	0.716	10	49.1

**Table 2:** *Pogona* variable importance.

	Variable	Permutation importance
*Pogona barbata*	Bio 12	45.9
	Bio 05	34.2
	Bio 17	19.2
	Bio 06	0.7
	Bio 01	0
	Bio 16	0
*Pogona henrylawsoni*	Bio 05	41.8
	Bio 12	36
	Bio 17	20.3
	Bio 16	1.9
	Bio 01	0
	Bio 06	0
*Pogona microlepidota*	Bio 01	86.9
	Bio 05	10.7
	Bio 17	1.8
	Bio 16	0.7
	Bio 06	0
	Bio 12	0
*Pogona minor*	Bio 01	37.4
	Bio 17	32.4
	Bio 16	19
	Bio 06	6.3
	Bio 12	4.7
	Bio 05	0.1
*Pogona nullarbor*	Bio 05	55.2
	Bio 12	42.5
	Bio 16	1.7
	Bio 17	0.7
	Bio 01	0
	Bio 06	0
*Pogona vitticeps*	Bio 12	50.4
	Bio 17	22.3
	Bio 05	17.3
	Bio 06	4.5
	Bio 16	4.4
	Bio 01	1.1

When compared with the model that incorporated NDVI, the accuracy metrics were nearly identical (Table S1), and the ranking of climatic variable results was very similar (Table S2). The main differences were switches in rank between two variables with close permutation of importance values, for example, *P. minor* swapped the ranking of annual temperature and precipitation during the driest quarter (Table S2). For most species, with the exception of *P. henrylawsoni*, NDVI ranked as fairly unimportant when predicting suitable habitat.

Habitat was predicted as suitable for *P. barbata* along the east (Fig. 3B); however, there were a few isolated regions predicted as suitable where *P. barbata* is not known to occur. These areas included Tasmania, central Australia, and the southwest region of Western Australia. Suitable habitat was predicted along the south coast, but there was no apparent habitat connection between the southwest and southeast corners. Future 2070 models predicted a reduction in suitable habitat for *P. barbata* primarily in the west region of their known range (Figs. 3C and 3D). The future 2070 RCP 8.5 model predicted substantially more habitat reduction, primarily in the northwest region of *P. barbata*’s range. All past models predicted very small areas of habitat gain and loss primarily along the western and northern regions of *P. barbata*’s range (Figs. 3E–3G). The Last Glacial Maximum model predicted a small amount of habitat gain along the southern coast, which minimally connects the southeast and southwest corners (Fig. 3F).

*Pogona henrylawsoni* had a predicted range of suitable habitat in the area they are found today (Fig. 4B). Additionally, there were small isolated patches of predicted habitat in areas they are not known to occur, primarily in the central northwest region of Australia. Future 2070 models predicted suitable habitat increase which would connect the known range of *P. henrylawsoni* with the isolated patch of suitable habitat in the west; however, this connection is much weaker in the 2070 RCP 2.6 model (Figs. 4C and 4D). The future 2070 RCP 8.5 model predicted substantially more suitable habitat and showed expansion across the majority of the north and throughout a large portion of the central east. The mid-Holocene model predicted very little suitable habitat, and the Last Glacial Maximum model predicted no suitable habitat within the known range for *P. henrylawsoni* (Figs. 4E and 4F). The Last Interglacial model predicted suitable habitat in the northeast; however, this is well beyond the bounds of *P. henrylawsoni*’s modern range (Fig. 4G).

Predicted suitable habitat for *P. microlepidota* was along the northern coast of Australia (Fig. 5B); however, *P. microlepidota* is only known to the western portion of this prediction. Future 2070 models predicted habitat expansion further inland, and the results of the 2070 RCP 8.5 model were slightly more extensive (Figs. 5C and 5D). The mid-Holocene model predicted a slight loss in suitable habitat (Fig. 5E), the Last Glacial Maximum model predicted massive loss with suitable habitat restricted to the coastal region of *P. microlepidota*’s known range (Fig. 5F), and the Last Interglacial model predicted a slight gain in suitable habitat (Fig. 5G).

Suitable habitat for *P. minor* was predicted throughout most of Western Australia and included regions slightly beyond the known range, such as the Nullarbor and the eastern interior of Australia (Fig. 6B). The future 2070 RCP 2.6 model predicted minimal loss of suitable habitat in the north and small patches in the interior of *P. minor*’s range (Fig. 6C), but the future 2070 RCP 8.5 predicted a greater loss (Fig. 6D). All past models predicted suitable habitat expansion to the north and east (Figs. 6E–6G). The mid-Holocene model predicted the least expansion, while the Last Glacial Maximum predicted the most. The mid-Holocene model also predicted a slight loss in suitable habitat in the central and eastern regions of *P. minor*’s range, while the Last Interglacial model predicted habitat loss in the west region.

*Pogona nullarbor* was predicted to occur in the Nullarbor Plain (Fig. 7B). Additionally, there were a few isolated patches of predicted suitable habitat that fell outside of *P. nullarbor*’s range in the west, east, and on Tasmania. Future 2070 models predicted habitat loss in the north, and the 2070 RCP 8.5 model predicted greater loss (Figs. 7C and 7D). All past models predicted suitable habitat gain expanding northward from *P. nullarbor*’s modern range (Figs. 7E–7G). The mid-Holocene model predicted the least amount of habitat gain, and the Last Glacial Maximum model predicted a small reduction in habitat along the south cost.

Habitat was predicted as suitable throughout *P. vitticeps*’ known range, with the central region being considered highly suitable (Fig. 8B). Additionally, the model predicted suitable habitat well beyond the known range of *P. vitticeps*, expanding into the west. Both future 2070 models predicted a reduction in suitable habitat in the north; however, the 2070 RCP 8.5 model predicted substantially more habitat loss (Figs. 8C and 8D). The mid-Holocene model predicted a range of suitable habitat fairly similar to the modern prediction with the greatest amount of habitat gain in the northwest region (Fig. 8E). Both the Last Glacial Maximum and Last Interglacial model predicted habitat loss in the northern portion of *P. vitticeps*’ known range; however, the Last Interglacial model also predicted an elongated strip of habitat gain to the northeast of *P. vitticep*’s modern range (Figs. 8F and 8G). There was a substantial gain in habitat to the west in all past models; however, this is beyond the known range of *P. vitticeps*.

For each species of *Pogona*, the current predicted habitat map closely matched with that of the NDVI model (Fig. S1). The only species to show much of a difference was *P. henrylawsoni* (Fig. 4B; Fig. S1B). The predicted habitat range for *P. henrylawsoni* was more restricted and only included a portion of their known range when the NDVI variable was included. When comparing the CCSM4 GCM output with the MIROC and MPI ESM output, the results were similar for *P. barbata*, *P. minor*, and *P. vitticeps* (Figs. S2–S4). When examining the known range and surrounding areas for these three species, slight differences were observed such as areas of greater or less habitat gain/loss and shifts in isolated patches of habitat gain/loss. When comparing models for *P. henrylawsoni*, *P. microlepidota*, and *P. nullarbor*, results were also similar; however, a few projections predicted very different results (Figs. S5–S7). When comparing mid-Holocene output for *P. henrylawsoni*, both MIROC and MPI ESM predicted substantially less habitat loss (Fig. 4E; Figs. S5C and S5G). The future 2070 RCP 8.5 MIROC ESM output for *P. microlepidota* predicted habitat loss rather then habitat gain (Fig. 5D; Figs. S6B and S6F). The MPI ESM predicted almost no expansion in suitable habitat for *P. nullarbor* during the mid-Holocene, and MIROC ESM predicted a complete loss of all *P. nullarbor*’s suitable habitat with isolated habitat gains to the east and west during the Last Glacial Maximum (Figs. 7E and 7F; Figs. S7D and S7G).

## Discussion

The model’s ability to predict areas of suitable habitat is not a perfect match with the known distribution of several species. For *P. vitticeps*, *P. minor*, and *P. microlepidota* suitable habitat is predicted beyond their known range. It is possible the climatic conditions are suitable for *P. vitticeps* in the west, for *P. minor* in the central east and Nullarbor, and for *P. microlepidota* along the north coast; however, there could be other factors such as dispersal ability and niche competition that are keeping these species restricted to their known ranges. For *P. barbata* and *P. henrylawsoni*, suitable habitat is predicted in isolated areas where the species do not occur, and while it is possible these regions could be climatically suitable for them, there is no physical way for the species to reach these locations.

The AUC values for *P. barbata*, *P. minor*, and *P. vitticeps* are in the 0.7 range, which indicates a “good” model (Duan et al., 2014). *P. henrylawsoni*, *P. microlepidota*, and *P. nullarbor* have much higher AUC values, being in the 0.9 range, which indicates an “excellent” model. The extremely high AUC for *P. henrylawsoni*, *P. microlepidota*, and *P. nullarbor* could be due to the small sample size, and/or their very restricted distribution (Phillips et al., 2009); however, the resulting prediction is accurate since occurrence data for the species were well-sampled within their known habitat. A well-sampled dataset greatly reduces any bias associated with small sample sizes and is actually much better than models produced from very large, but poorly-sampled datasets (Bean, Stafford & Brashares, 2012). While current prediction models for *P. henrylawsoni*, *P. microlepidota*, and *P. nullarbor* corroborated Bean, Stafford & Brashares (2012), comparisons of future and past GCM projections (i.e., CCSM4, MIROC, and MPI) indicated more variance than models for the other three species, which included a greater number of sample points over larger areas. Consequently, uncertainty of future and past climate projections for *P. henrylawsoni*, *P. microlepidota*, and *P. nullarbor* should be considered, with a wider range of suitable habitats possible.

The results of the current model and the model including NDVI are similar, confirming that the climate variables are a good indicator for predicting *Pogona* species occurrences, and can be used independent of NDVI to project future and past occurrences. In the past, *P. barbata* is predicted to have had a similar range in suitable habitat as the predicted current range. The fossil record for small mammals suggests a wetter central south Australian coast between 15,000 and 30,000 years ago which connected the southeast and southwest regions of Australia (Lundelius, 1983), and our results show a small potential of habitat connection for *P. barbata* during this time. Our results predict minimal suitable habitat for *P. henrylawsoni* and *P. microlepidota* during the Last Glacial Maximum; however, based on genetic studies, *P. henrylawsoni* is estimated to have emerged ∼2.5 million years ago, and *P. microlepidota* is estimated to have emerged ∼1.3 million years ago (Chen et al., 2012). Given this discrepancy, the model could have potentially failed to recognize suitable habitat, the species’ tolerance might have changed, or species could have occurred in areas outside of the modern continental boundary of Australia. During the Last Glacial Maximum, sea level was lower which left the continental shelf of Australia largely exposed (Yokoyama et al., 2001), and it is possible species such as *P. microlepidota*, were persisting in these exposed regions, especially since our results show them restricted to the coast.

When examining the known ranges of *Pogona* species, *P. minor* and *P. nullarbor* are predicted to have had an expanded range of suitable habitat in the past, while *P. vitticeps* is predicted to have had a reduction in suitable habitat. If examining regions beyond the known range, *P. vitticeps* would have had substantial gains in suitable habitat in Western Australia; however, the likelihood of this species existing in the west is unknown. All predictions from the past models should be compared with the *Pogona* fossil record once possible. We are currently examining fossil *Pogona* along with other groups of agamids from Western Australia. Once our fossil specimens are identified to the species level, we will compare fossil localities with these past projections. Having actual fossil localities for species of *Pogona* could help improve the models and this information can potentially then be studied in other lizard groups, providing critical evidence for conservation efforts.

The future 2070 model output for each *Pogona* species are similar between the RCP 2.6 (best-case scenario where emissions are reduced) and the RCP 8.5 (worst-case scenario where emissions will increase); however, the worst-case model results are more intensified with substantially more habitat loss and gain. In the future, *P. henrylawsoni* and *P. microlepidota* are the only species predicted to experience a gain in suitable habitat; however, it is unknown whether these lizards will expand their range due to factors such as competition and rates of dispersal. Given the best-case scenario, *P. barbata* and *P. minor* are predicted to lose a minimal amount of suitable habitat, and while the predicted loss in habitat is more substantial in the worst-case scenario, these two species should be of least concern.

*Pogona vitticeps* may lose a fair amount of habitat, especially given the worst-case scenario. Based on all projections, the best climatic conditions for suitable habitat within *P. vitticeps*’ known range are those of the modern and mid-Holocene. Their area of suitable habitat is predicted to have increased over the past 120,000 years but will decline in the future as the climate warms. *P. nullarbor* already has a restricted range which will only reduce further over time given either scenario. In the past, *P. nullarbor*’s predicted habitat was greater; however, their habitat area is shown to be in a decline over the past 20,000 years. This declining trend is then predicted to continue into the future. Currently, there is no IUCN status for *P. nullarbor* or *P. vitticeps*, but *P. nullarbor* should at least be considered vulnerable and a conservation plan may need to be considered in the future. *P. vitticeps* has a large population and an extensive range today, and while the models predict a potentially large loss in suitable habitat, this species does not need to be considered for a conservation plan yet; however, populations should be monitored if there is a decline through the next several decades. Overall, the genus *Pogona* is expected to survive into the future, and diversity will not be greatly impacted as long as greenhouse gas emissions are not higher than the scenarios used in this study.

## Conclusions

MaxEnt is widely used for modeling potential habitat suitability during past, current, and future time periods, and has been used to model other lizard groups across the world. In this study, the genus *Pogona* was modeled to predict suitable habitat in the past, current, and future to gain an understanding of the different species’ potential responses to climate change. Models predicting areas of suitable habitat in the past should be compared to the *Pogona* fossil record once possible. If there is a difference between the fossil record and the predicted model, it could be due to a change in the lizard’s physiology as it adapts to the changing environment and climate, or it could be due to other factors such as niche partitioning. This evolutionary information would be of great value and could be used to improve the model’s ability to predict suitable habitat. Future models predict that *P. henrylawsoni* and *P. microlepidota* will gain suitable habitat, while the other four species are predicted to lose suitable habitat with *P. nullarbor* and *P. vitticeps* showing the most potential loss. *P. nullarbor* and *P. vitticeps* populations should be closely monitored for any signs of decline, and a conservation plan for *P. nullarbor* may need to be considered. The results of this study are only one set of potential habitat hypotheses and should be compared with other analyses not explored here such as molecular or phylogeographic data, population trends, extinctions of local populations, discovery of new populations, and additional modeling algorithms.

## Supplemental Information

10.7717/peerj.6128/supp-1 Supplemental Information 1 Result maps with the inclusion of NDVI. (A) *P. barbata* where highly suitable is > 41.83% of occurrences predicted, and low suitability is < 18.01% of occurrences predicted. (B) *P. henrylawsoni* where highly suitable is > 38.82% of occurrences predicted, and low suitability is < 2.75% of occurrences predicted. (C) *P. microlepidota* where highly suitable is > 16.08% of occurrences predicted, and low suitability is < 3.92% of occurrences predicted. (D) *P. minor* where highly suitable is > 21.96% of occurrences predicted, and low suitability is < 11.37% of occurrences predicted. (E) *P. nullarbor* where highly suitable is > 5.88% of occurrences predicted, and low suitability is < 1.18% of occurrences predicted. (F) *P. vitticeps* where highly suitable is > 39.68% of occurrences predicted, and low suitability is < 18.92% of occurrences predicted.

10.7717/peerj.6128/supp-2 Supplemental Information 2 MIROC and MPI result maps for *Pogona barbata*. (A) MIROC Future (2070 RCP 2.6) predicted change. (B) MIROC Future (2070 RCP 8.5) predicted change. (C) MIROC Past (mid-Holocene) predicted change. (D) MIROC Past (Last Glacial Maximum) predicted change. (E) MPI Future (2070 RCP 2.6) predicted change. (F) MPI Future (2070 RCP 8.5) predicted change. (G) MPI Past (mid-Holocene) predicted change. (H) MPI Past (Last Glacial Maximum) predicted change.

10.7717/peerj.6128/supp-3 Supplemental Information 3 MIROC and MPI result maps for *Pogona minor*. (A) MIROC Future (2070 RCP 2.6) predicted change. (B) MIROC Future (2070 RCP 8.5) predicted change. (C) MIROC Past (mid-Holocene) predicted change. (D) MIROC Past (Last Glacial Maximum) predicted change. (E) MPI Future (2070 RCP 2.6) predicted change. (F) MPI Future (2070 RCP 8.5) predicted change. (G) MPI Past (mid-Holocene) predicted change. (H) MPI Past (Last Glacial Maximum) predicted change.

10.7717/peerj.6128/supp-4 Supplemental Information 4 MIROC and MPI result maps for *Pogona vitticeps*. (A) MIROC Future (2070 RCP 2.6) predicted change. (B) MIROC Future (2070 RCP 8.5) predicted change. (C) MIROC Past (mid-Holocene) predicted change. (D) MIROC Past (Last Glacial Maximum) predicted change. (E) MPI Future (2070 RCP 2.6) predicted change. (F) MPI Future (2070 RCP 8.5) predicted change. (G) MPI Past (mid-Holocene) predicted change. (H) MPI Past (Last Glacial Maximum) predicted change.

10.7717/peerj.6128/supp-5 Supplemental Information 5 MIROC and MPI result maps for *Pogona henrylawsoni*. (A) MIROC Future (2070 RCP 2.6) predicted change. (B) MIROC Future (2070 RCP 8.5) predicted change. (C) MIROC Past (mid-Holocene) predicted change. (D) MIROC Past (Last Glacial Maximum) predicted change. (E) MPI Future (2070 RCP 2.6) predicted change. (F) MPI Future (2070 RCP 8.5) predicted change. (G) MPI Past (mid-Holocene) predicted change. (H) MPI Past (Last Glacial Maximum) predicted change.

10.7717/peerj.6128/supp-6 Supplemental Information 6 MIROC and MPI result maps for *Pogona microlepidota.*. (A) MIROC Future (2070 RCP 2.6) predicted change. (B) MIROC Future (2070 RCP 8.5) predicted change. (C) MIROC Past (mid-Holocene) predicted change. (D) MIROC Past (Last Glacial Maximum) predicted change. (E) MPI Future (2070 RCP 2.6) predicted change. (F) MPI Future (2070 RCP 8.5) predicted change. (G) MPI Past (mid-Holocene) predicted change. (H) MPI Past (Last Glacial Maximum) predicted change.

10.7717/peerj.6128/supp-7 Supplemental Information 7 MIROC and MPI result maps for *Pogona nullarbor*. (A) MIROC Future (2070 RCP 2.6) predicted change. (B) MIROC Future (2070 RCP 8.5) predicted change. (C) MIROC Past (mid-Holocene) predicted change. (D) MIROC Past (Last Glacial Maximum) predicted change. (E) MPI Future (2070 RCP 2.6) predicted change. (F) MPI Future (2070 RCP 8.5) predicted change. (G) MPI Past (mid-Holocene) predicted change. (H) MPI Past (Last Glacial Maximum) predicted change.

10.7717/peerj.6128/supp-8 Supplemental Information 8 *Pogona* accuracy metrics with the inclusion of NDVI.

10.7717/peerj.6128/supp-9 Supplemental Information 9 *Pogona* variable importance with the inclusion of NDVI.

10.7717/peerj.6128/supp-10 Supplemental Information 10 Train point raw data 1. Train longitude and latitude coordinates for *Pogona* species: *P. barbata*, *P. henrylawsoni*, *P. microlepidota*, *P. minor*, *P. nullarbor*, and *P. vitticeps*.

10.7717/peerj.6128/supp-11 Supplemental Information 11 Test point raw data 2. Test longitude and latitude coordinates for *Pogona* species: *P. barbata*, *P. henrylawsoni*, *P. microlepidota*, *P. minor*, *P. nullarbor*, and *P. vitticeps*.

## Additional Information and Declarations

### Data Availability

The following information was supplied regarding data availability: Rej, Julie (2018): ndvi_of_Australia.asc. figshare. Dataset. https://doi.org/10.6084/m9.figshare.7115441.v1.
