# Supplementary figures and images for: Niche modeling for the genus Pogona (Squamata: Agamidae) in Australia: predicting past (late Quaternary) and future (2070) areas of suitable habitat
Source: PeerJ. 2018 Dec 17;6:e6128. doi: 10.7717/peerj.6128 (PMC6301283; doi:10.7717/peerj.6128)

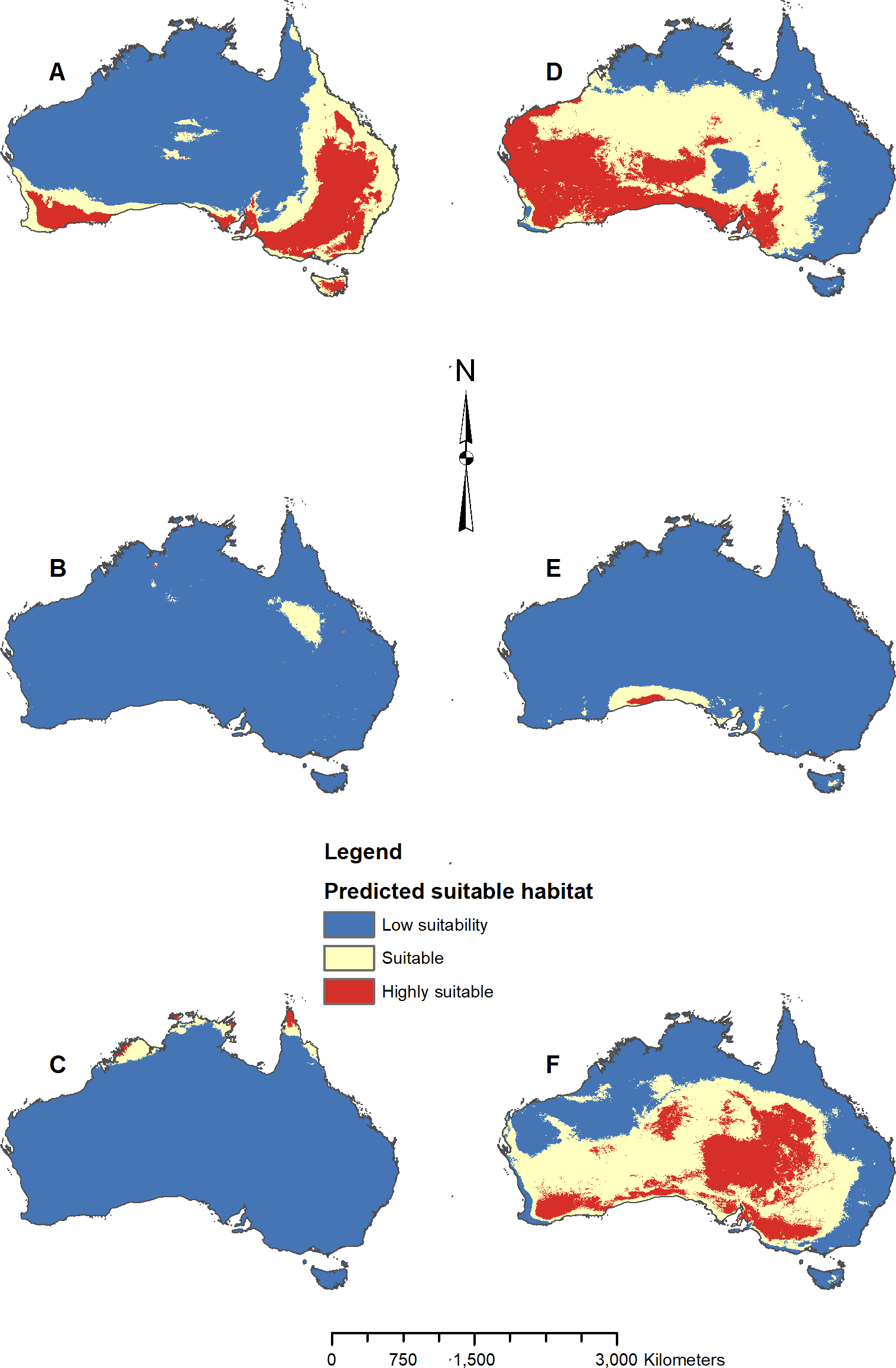

Supplement: Supplemental Information 1 — (A) P. barbata where highly suitable is > 41.83% of occurrences predicted, and low suitability is < 18.01% of occurrences predicted. (B) P. henrylawsoni where highly suitable is > 38.82% of occurrences predicted, and low suitability is < 2.75% of occurrences predicted. (C) P. microlepidota where highly suitable is > 16.08% of occurrences predicted, and low suitability is < 3.92% of occurrences predicted. (D) P. minor where highly suitable is > 21.96% of occurrences predicted, and low suitability is < 11.37% of occurrences predicted. (E) P. nullarbor where highly suitable is > 5.88% of occurrences predicted, and low suitability is < 1.18% of occurrences predicted. (F) P. vitticeps where highly suitable is > 39.68% of occurrences predicted, and low suitability is < 18.92% of occurrences predicted. [file peerj-06-6128-s001.png]

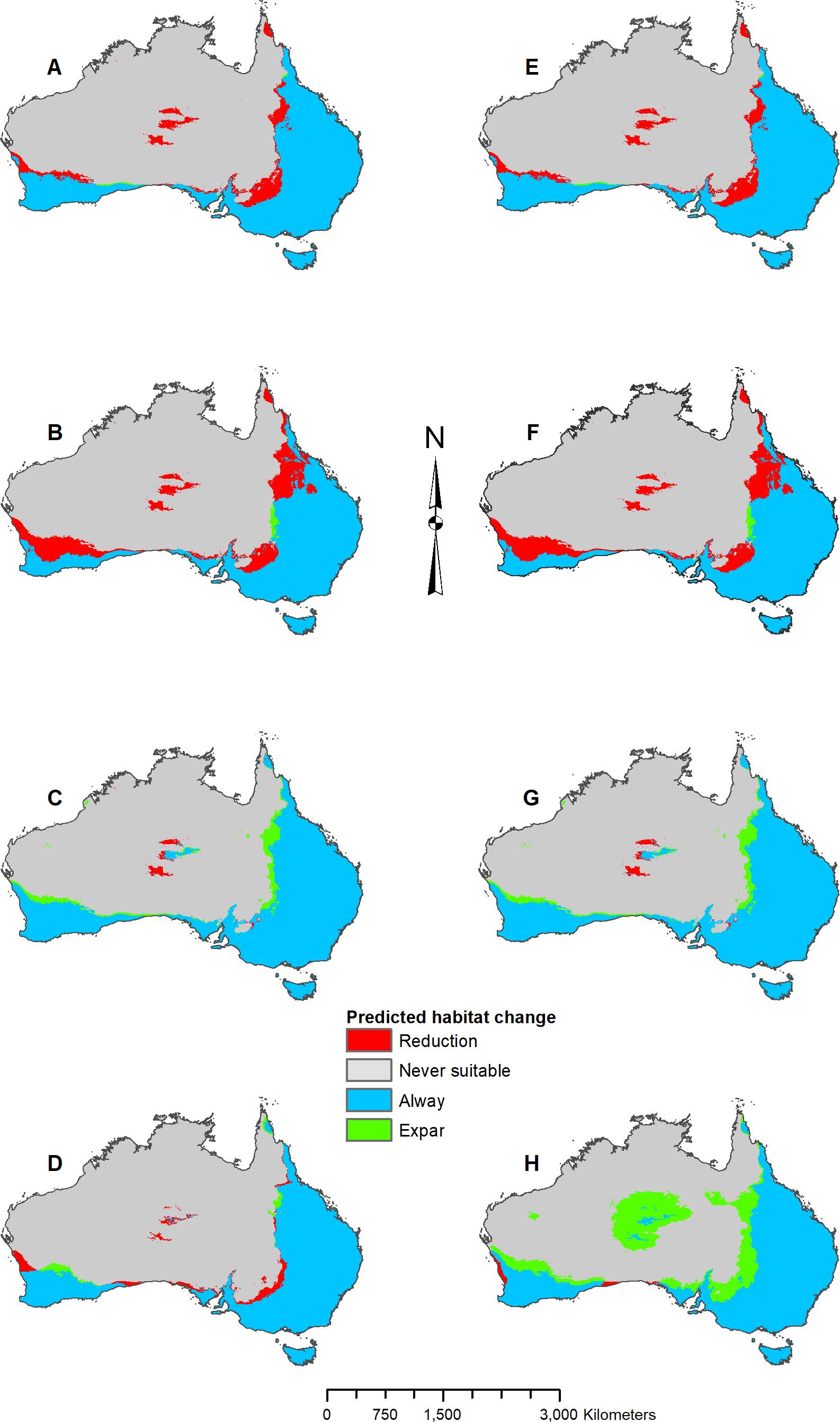

Supplement: Supplemental Information 2 — (A) MIROC Future (2070 RCP 2.6) predicted change. (B) MIROC Future (2070 RCP 8.5) predicted change. (C) MIROC Past (mid-Holocene) predicted change. (D) MIROC Past (Last Glacial Maximum) predicted change. (E) MPI Future (2070 RCP 2.6) predicted change. (F) MPI Future (2070 RCP 8.5) predicted change. (G) MPI Past (mid-Holocene) predicted change. (H) MPI Past (Last Glacial Maximum) predicted change. [file peerj-06-6128-s002.png]

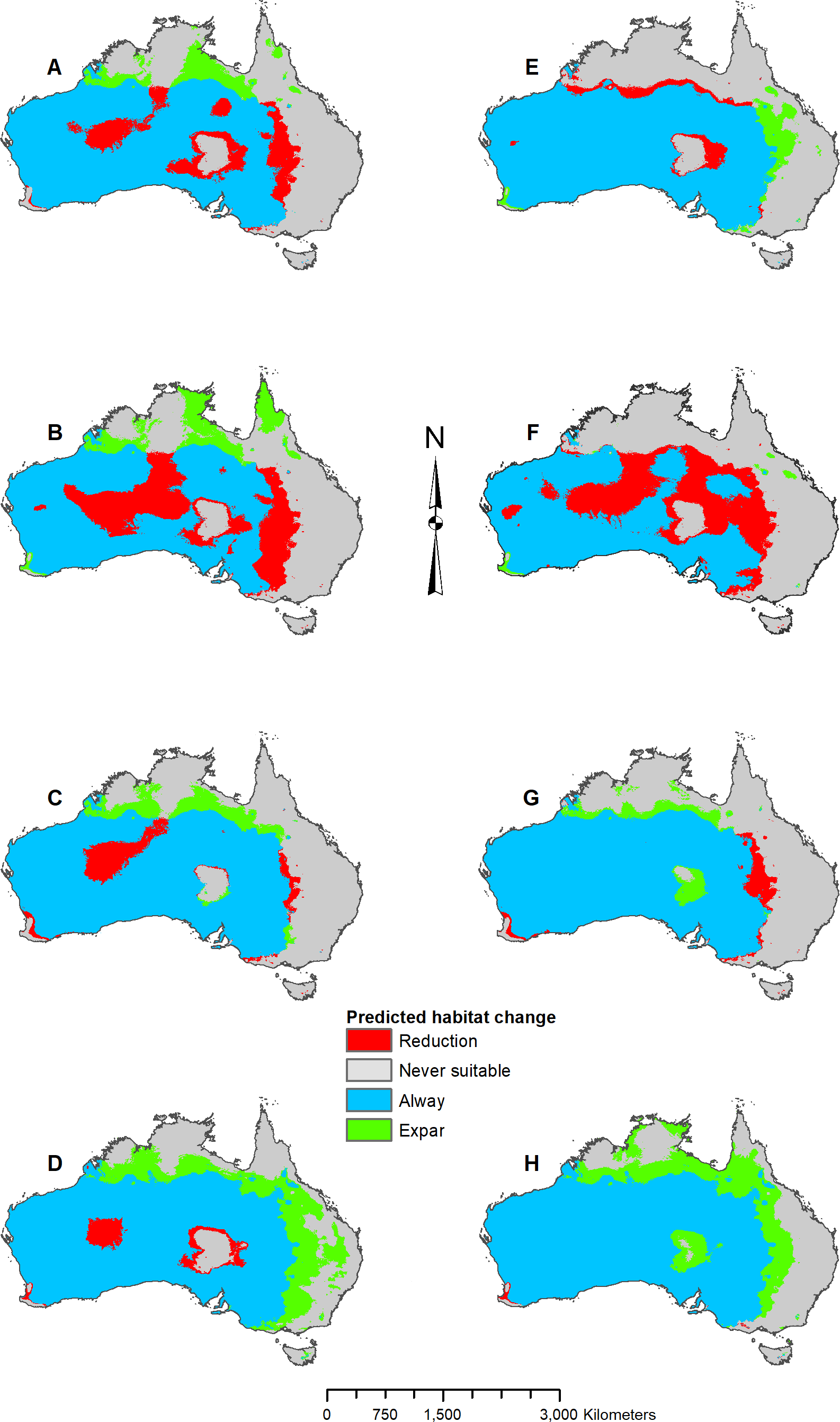

Supplement: Supplemental Information 3 — (A) MIROC Future (2070 RCP 2.6) predicted change. (B) MIROC Future (2070 RCP 8.5) predicted change. (C) MIROC Past (mid-Holocene) predicted change. (D) MIROC Past (Last Glacial Maximum) predicted change. (E) MPI Future (2070 RCP 2.6) predicted change. (F) MPI Future (2070 RCP 8.5) predicted change. (G) MPI Past (mid-Holocene) predicted change. (H) MPI Past (Last Glacial Maximum) predicted change. [file peerj-06-6128-s003.png]

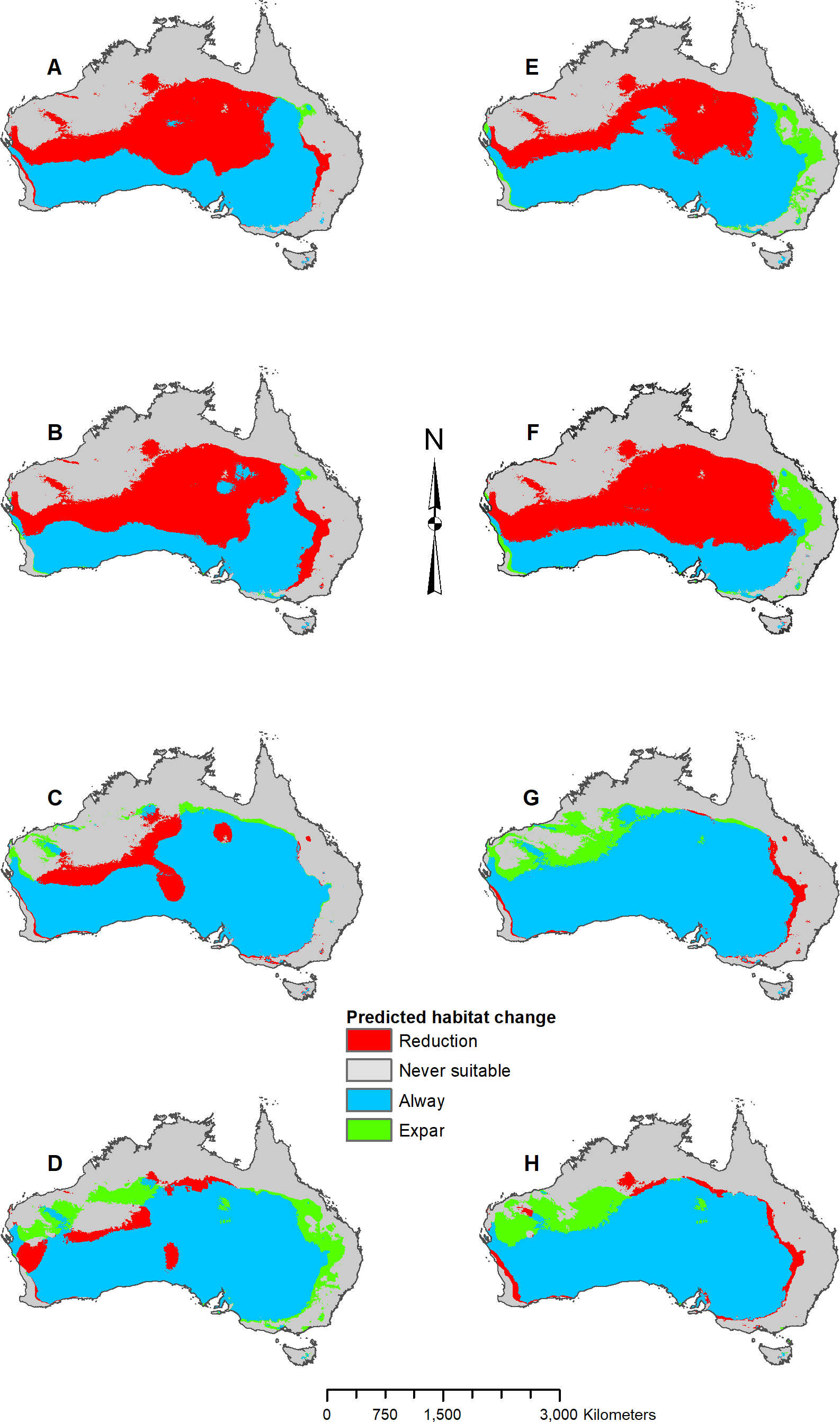

Supplement: Supplemental Information 4 — (A) MIROC Future (2070 RCP 2.6) predicted change. (B) MIROC Future (2070 RCP 8.5) predicted change. (C) MIROC Past (mid-Holocene) predicted change. (D) MIROC Past (Last Glacial Maximum) predicted change. (E) MPI Future (2070 RCP 2.6) predicted change. (F) MPI Future (2070 RCP 8.5) predicted change. (G) MPI Past (mid-Holocene) predicted change. (H) MPI Past (Last Glacial Maximum) predicted change. [file peerj-06-6128-s004.png]

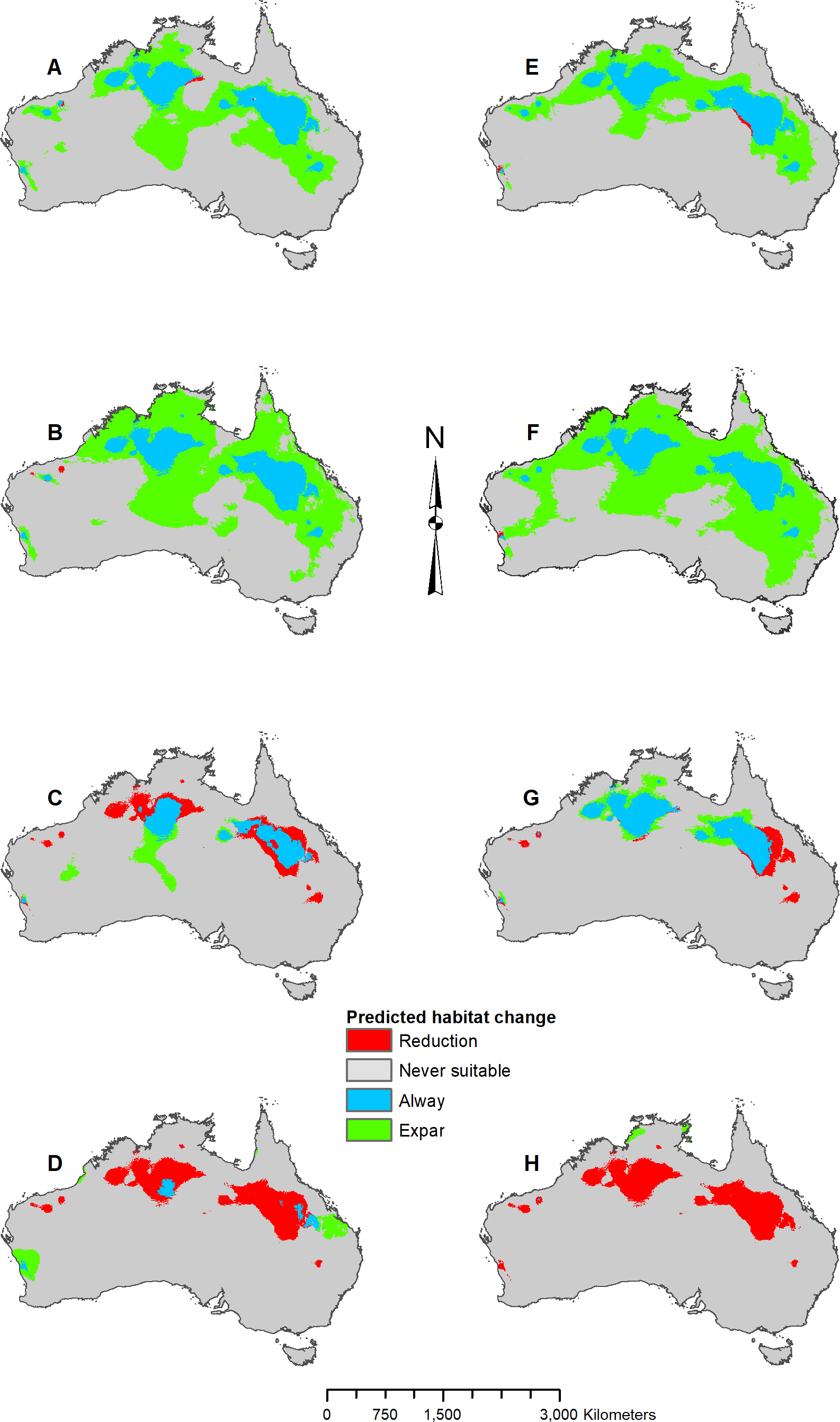

Supplement: Supplemental Information 5 — (A) MIROC Future (2070 RCP 2.6) predicted change. (B) MIROC Future (2070 RCP 8.5) predicted change. (C) MIROC Past (mid-Holocene) predicted change. (D) MIROC Past (Last Glacial Maximum) predicted change. (E) MPI Future (2070 RCP 2.6) predicted change. (F) MPI Future (2070 RCP 8.5) predicted change. (G) MPI Past (mid-Holocene) predicted change. (H) MPI Past (Last Glacial Maximum) predicted change. [file peerj-06-6128-s005.png]

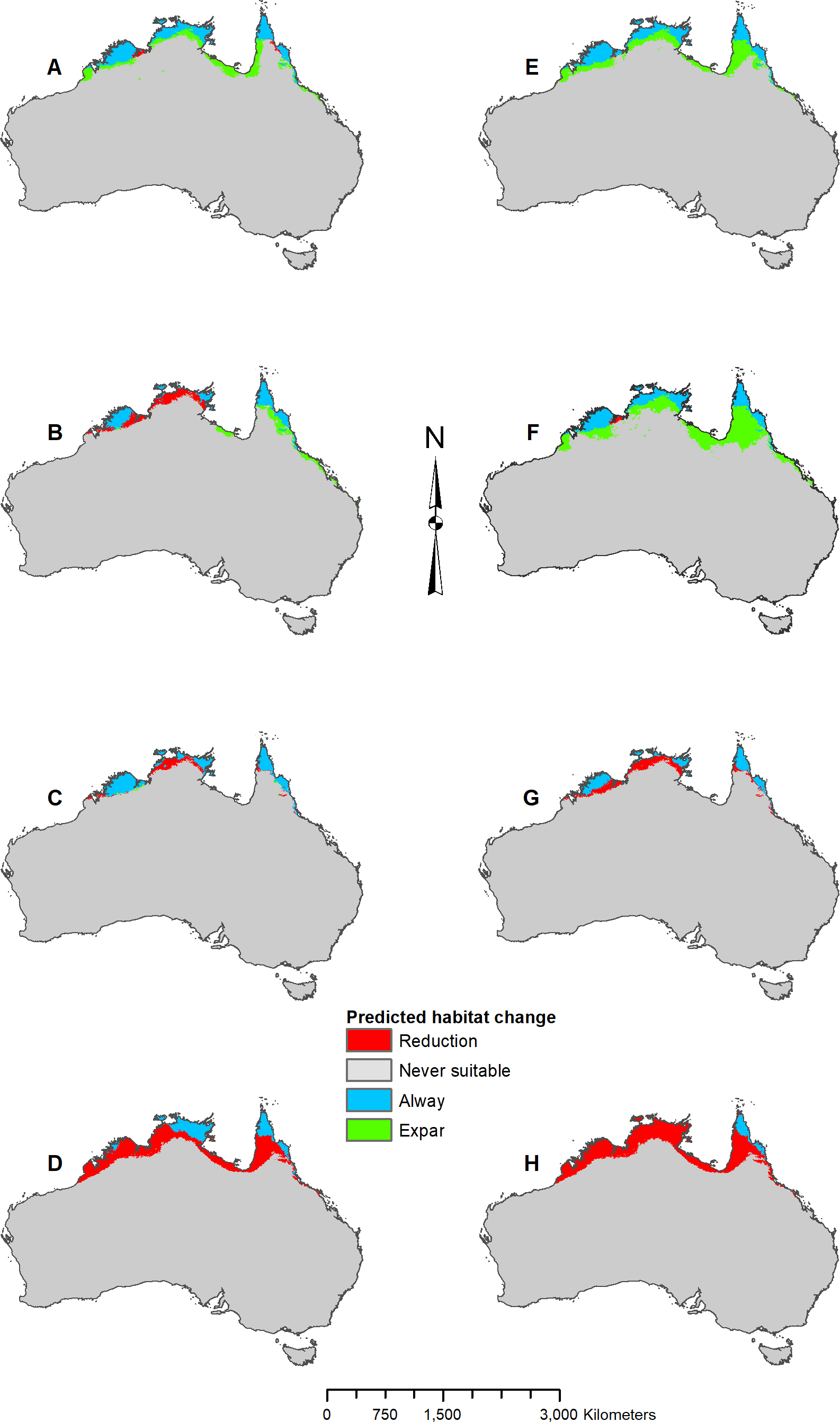

Supplement: Supplemental Information 6 — (A) MIROC Future (2070 RCP 2.6) predicted change. (B) MIROC Future (2070 RCP 8.5) predicted change. (C) MIROC Past (mid-Holocene) predicted change. (D) MIROC Past (Last Glacial Maximum) predicted change. (E) MPI Future (2070 RCP 2.6) predicted change. (F) MPI Future (2070 RCP 8.5) predicted change. (G) MPI Past (mid-Holocene) predicted change. (H) MPI Past (Last Glacial Maximum) predicted change. [file peerj-06-6128-s006.png]

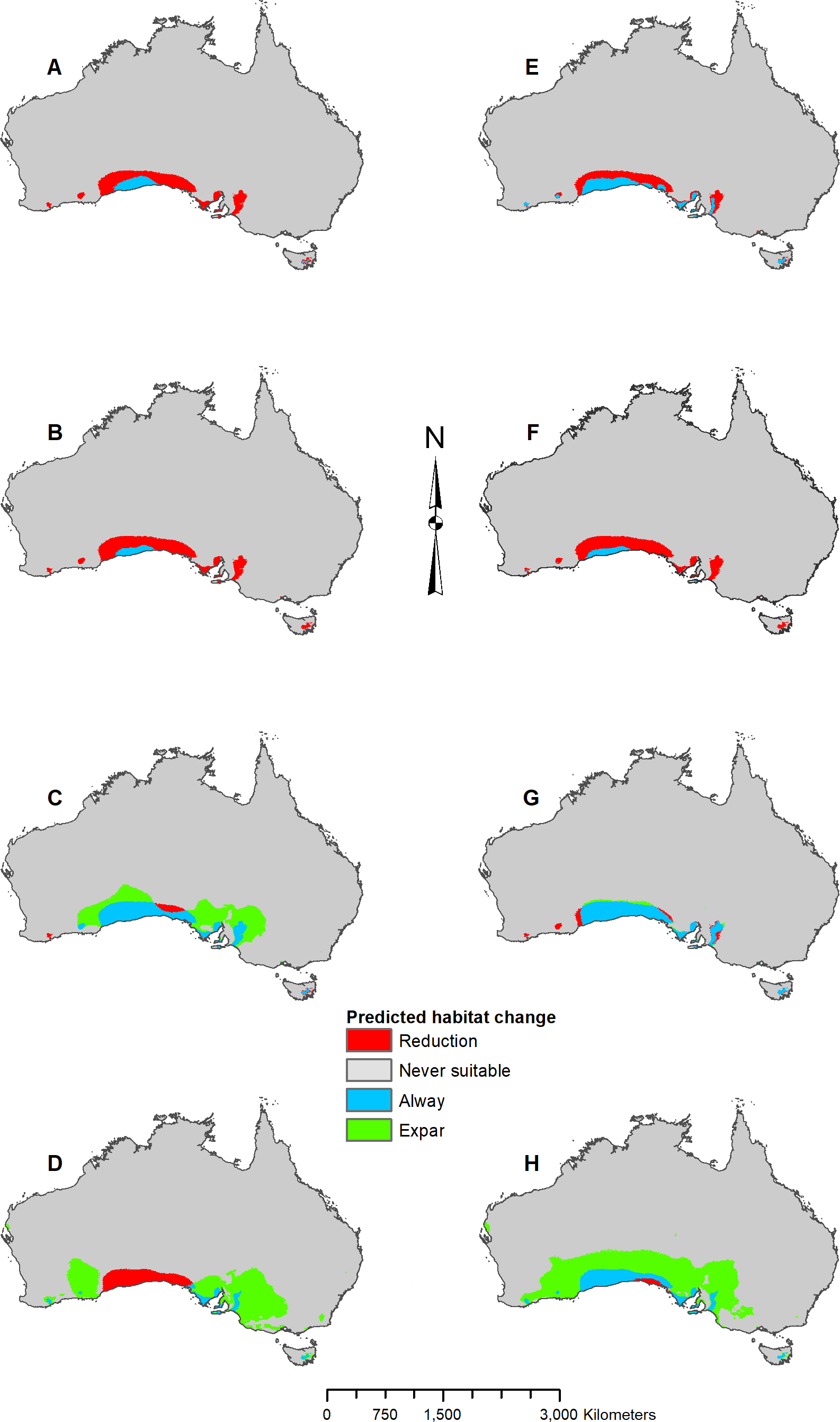

Supplement: Supplemental Information 7 — (A) MIROC Future (2070 RCP 2.6) predicted change. (B) MIROC Future (2070 RCP 8.5) predicted change. (C) MIROC Past (mid-Holocene) predicted change. (D) MIROC Past (Last Glacial Maximum) predicted change. (E) MPI Future (2070 RCP 2.6) predicted change. (F) MPI Future (2070 RCP 8.5) predicted change. (G) MPI Past (mid-Holocene) predicted change. (H) MPI Past (Last Glacial Maximum) predicted change. [file peerj-06-6128-s007.png]
