# Supplementary material for: Niche modeling for the genus Pogona (Squamata: Agamidae) in Australia: predicting past (late Quaternary) and future (2070) areas of suitable habitat
Source: PeerJ. 2018 Dec 17;6:e6128. doi: 10.7717/peerj.6128 (PMC6301283; doi:10.7717/peerj.6128)
Supplement: Supplemental Information 8 [file peerj-06-6128-s008.docx]

|  | **Training AUC** | **Testing AUC** | **Omission** | **Commission** |
| --- | --- | --- | --- | --- |
| ***Pogona barbata*** | 0.774 | 0.773 | 1.3% | 40.2% |
| ***Pogona henrylawsoni*** | 0.975 | 0.984 | 8.3% | 8.2% |
| ***Pogona microlepidota*** | 0.986 | 0.962 | 6.2% | 6.1% |
| ***Pogona minor*** | 0.773 | 0.765 | 10% | 57.3% |
| ***Pogona nullarbor*** | 0.989 | 0.984 | 10% | 2.5% |
| ***Pogona vitticeps*** | 0.739 | 0.717 | 10% | 50.7% |
