# Supplementary material for: Niche modeling for the genus Pogona (Squamata: Agamidae) in Australia: predicting past (late Quaternary) and future (2070) areas of suitable habitat
Source: PeerJ. 2018 Dec 17;6:e6128. doi: 10.7717/peerj.6128 (PMC6301283; doi:10.7717/peerj.6128)
Supplement: Supplemental Information 9 [file peerj-06-6128-s009.docx]

|  | **Variable** | **Permutation importance** |
| --- | --- | --- |
| ***Pogona barbata*** | Bio 12 | 47.4 |
|  | Bio 05 | 34.9 |
|  | Bio 17 | 17 |
|  | Bio 06 | 0.7 |
|  | Bio 01 | 0 |
|  | Bio 16 | 0 |
|  | NDVI | 0 |
| ***Pogona henrylawsoni*** | Bio 12 | 37.3 |
|  | Bio 05 | 30.1 |
|  | NDVI | 17 |
|  | Bio 17 | 13.9 |
|  | Bio 16 | 1.6 |
|  | Bio 01 | 0 |
|  | Bio 06 | 0 |
| ***Pogona microlepidota*** | Bio 01 | 85.2 |
|  | Bio 05 | 12 |
|  | Bio 17 | 1.9 |
|  | Bio 16 | 0.7 |
|  | NDVI | 0.2 |
|  | Bio 06 | 0 |
|  | Bio 12 | 0 |
| ***Pogona minor*** | Bio 17 | 32.7 |
|  | Bio 01 | 29.6 |
|  | Bio 16 | 17.9 |
|  | NDVI | 9.2 |
|  | Bio 12 | 5.7 |
|  | Bio 06 | 4.5 |
|  | Bio 05 | 0.5 |
| ***Pogona nullarbor*** | Bio 05 | 60.8 |
|  | Bio 12 | 35.3 |
|  | Bio 17 | 1.5 |
|  | NDVI | 1.3 |
|  | Bio 16 | 1.2 |
|  | Bio 01 | 0 |
|  | Bio 06 | 0 |
| ***Pogona vitticeps*** | Bio 12 | 48.2 |
|  | Bio 17 | 19.4 |
|  | Bio 05 | 14.8 |
|  | Bio 06 | 8.5 |
|  | Bio 01 | 4.8 |
|  | Bio 16 | 4.3 |
|  | NDVI | 0 |
